# Ultra-high field neuroimaging reveals fine-scale processing for 3D perception

**DOI:** 10.1523/JNEUROSCI.0065-21.2021

**Published:** 2021-08-19

**Authors:** Adrian K. T. Ng, Ke Jia, Nuno R. Goncalves, Elisa Zamboni, Valentin Kemper, Rainer Goebel, Andrew E. Welchman, Zoe Kourtzi

**Affiliations:** 1 Department of Psychology, University of Cambridge, Cambridge, CB2 3EB, UK; 2 Department of Cognitive Neuroscience, Faculty of Psychology and Neuroscience, Maastricht University, Maastricht, 6200 MD, The Netherlands; 3 Department of Cognitive Neuroscience, Maastricht Brain Imaging Centre, Maastricht University, Maastricht, 6220 MD, The Netherlands; 4 Department of Industrial and Manufacturing Systems Engineering, The University of Hong Kong, Hong Kong SAR, China

## Abstract

Binocular disparity provides critical information about three-dimensional (3D) structure to support perception and action. The past decade has seen significant progress in uncovering human brain areas engaged in the processing of binocular disparity signals. Yet, the fine-scale brain processing underlying 3D perception remains unknown. Here, we use ultra-high field (7T) functional imagining at sub-millimetre resolution to examine fine-scale BOLD-fMRI signals involved in 3D perception. In particular, we sought to interrogate the local circuitry involved in disparity processing by sampling fMRI responses at different positions relative to the cortical surface (i.e., across cortical depths corresponding to layers). We test for representations related to 3D perception by presenting participants (male and female, N = 8) with stimuli that enable stable stereoscopic perception (i.e., correlated random dot stereograms: RDS) vs. those that do not (i.e., anti-correlated RDS). Using multi-voxel pattern analysis (MVPA), we demonstrate cortical depth-specific representations in area V3A and V7 as indicated by stronger pattern responses for correlated than anti-correlated stimuli in upper than deeper layers. Examining informational connectivity, we find higher feedforward layer-to-layer connectivity for correlated than anti-correlated stimuli between V3A and V7. Further, we observe disparity-specific feedback from V3A to V1 and from V7 to V3A. Our findings provide evidence for the role of V3A as a key nexus for disparity processing that is implicated in feedforward and feedback signals related to the perceptual estimation of 3D structure.

## Introduction

Binocular vision affords humans two slightly different views of the world (i.e., binocular disparity) from which the brain can triangulate the structure of the environment. The process of inferring 3D structure from differences in the signals registered in the two eyes is the result of an unknown series of computations that starts in primary visual cortex—the first point of integration of signals from the two eyes. Human neuroimaging studies have revealed widespread responses to binocular signals in occipito-parietal regions (Bridge and Parker, 2007; Welchman, 2016) with the strongest modulation of brain activity by binocular signals in areas V3A and V7 (Backus et al., 2001; Tsao et al., 2003; Preston et al., 2008; Minini et al., 2010; Cottereau et al., 2011; Goncalves et al., 2015; Rideaux and Welchman, 2019; Liu et al., 2020). There is evidence for local clustering of disparity-selective organization in area V3A (Goncalves et al., 2015; Parker et al., 2017; Tootell and Nasr, 2017; Nasr and Tootell, 2020), as well as V2 and V3 (Nasr and Tootell, 2018). These findings indicate parts of the dorsal visual cortex that are heavily involved in processing disparity signals. However, the fine-scale processing and interactions among these regions that support our ability to perceive 3D structure remain largely unknown. Our understanding of how disparity signals are processed within and between different cortical regions is limited: with some evidence suggesting local recurrent processing (Tanabe and Cumming, 2014) while other studies pointing to interactions between areas (Cottereau et al., 2014).

**Figure 1:**
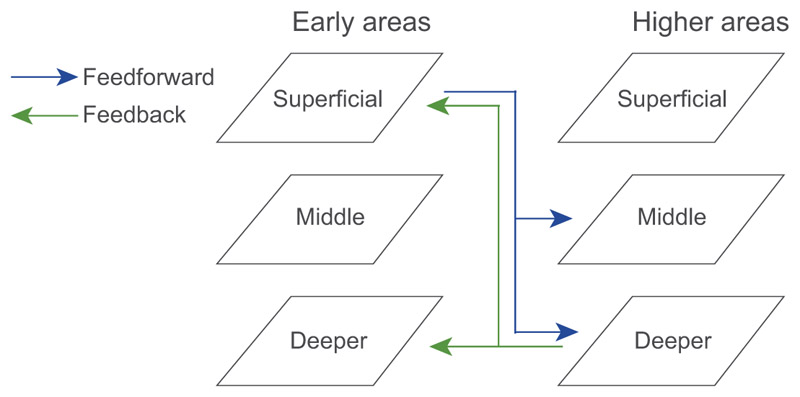
fMRI circuits across cortical depth. Schematic representation of feedforward (superficial – middle layers; blue) and feedback (deeper – deeper layers; green) anatomical connectivity between early and higher visual areas based on known anatomical circuits.

Here, we capitalize on recent advances in ultra-high field (UHF) imaging to examine the local circuitry for disparity processing at finer scale in the human brain. UHF imaging affords the sub-millimetre resolution necessary to examine functional activation within a visual area at different positions relative to the cortical surface (i.e., cortical depth) in a non-invasive manner (for review, see Goense et al., 2016; Lawrence et al., 2019b). This allows us to test functional connectivity across cortical layers based on known anatomical laminar circuits from electrophysiological studies (Fig. 1; for review, see Self et al., 2019). Specifically, sensory input is known to enter the visual cortex from the thalamus at the level of the middle layer (layer 4) (Hubel and Wiesel, 1972; Blasdel and Lund, 1983), while output information is fed forward from superficial layers (layer 2/3) (Livingstone and Hubel, 1984). Feedback information is exchanged primarily between deeper layers (layer 5/6) and from deeper to superficial layers (Rockland and Pandya, 1979). This circuitry motif has been implicated in a range of visual computations revealed through neurophysiological studies (e.g., Self et al., 2013) and human fMRI studies that discern signals from different cortical depths to infer feedforward vs. feedback processing (e.g., Lawrence et al., 2019a; Jia et al., 2020; Zamboni et al., 2020).

**Figure 2:**
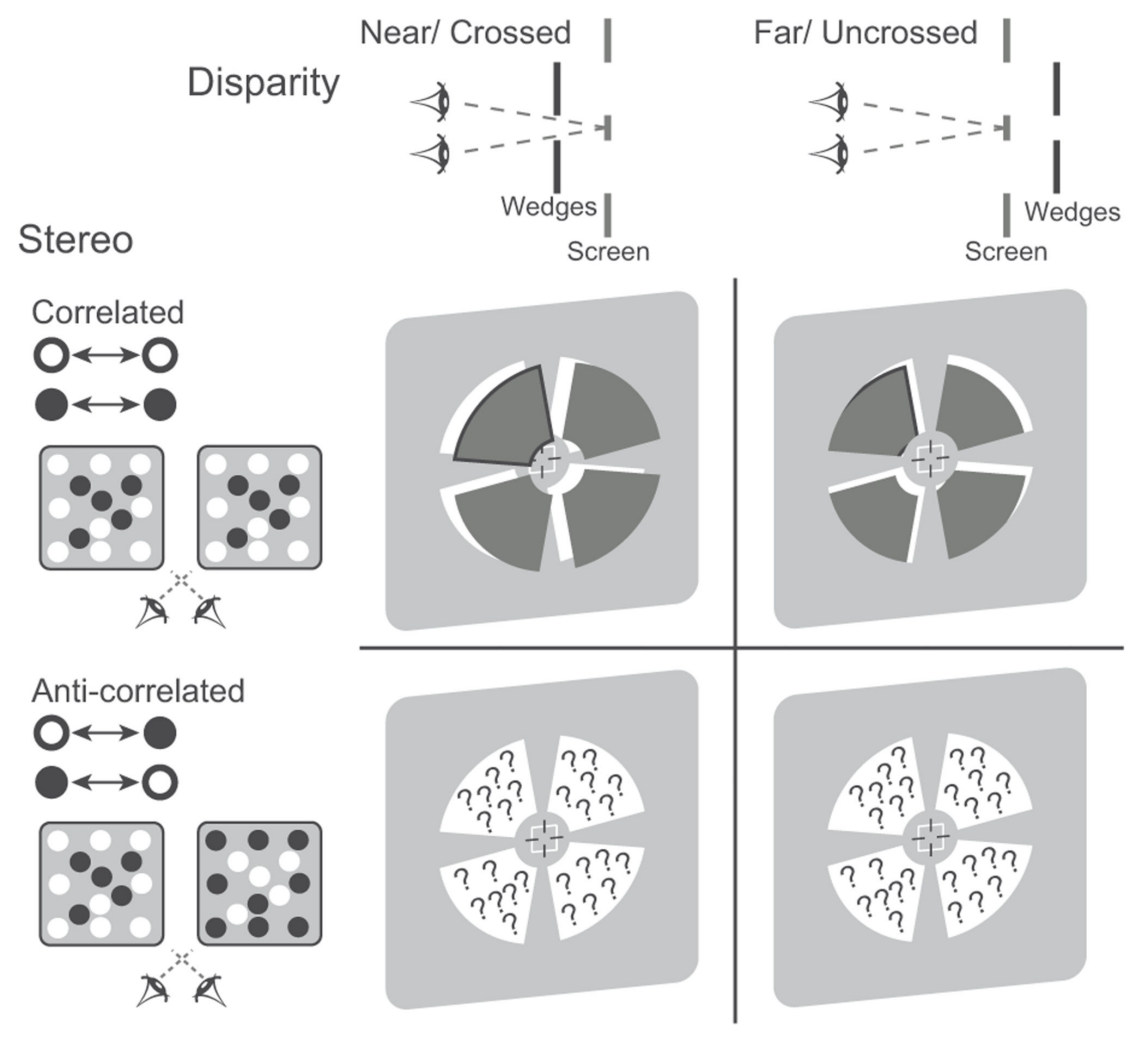
Schematic illustration of the stimuli and experimental conditions. (A) A simplified schematic representation of RDSs. Correlated RDS stimuli were created by showing dots comprising the stimulus presented at the same contrast in both eyes (e.g., a black dot in the left eye matched a black dot in the right eye). The same disparity configurations were rendered in the anti-correlated RDS, where the contrasts of dots in the two eyes were reversed (e.g., a white dot in one eye matched a black dot in the other). This anti-correlated stimulus does not evoke a reliable disparity-defined impression of 3D structure (stereoscopic perception). (B) A schematic representation of the disparity-defined 3D structure of the stimuli. The grey lines represent the fixation plane of the display screen where observers were instructed to fixate their eyes (zero disparity). One of two perceived depth configurations was presented on each trial: diagram on the left shows the “crossed” disparity configurations in which the wedges (represented by black lines) were closer to the observer than the fixation plane (i.e., disparity near), and diagram on the right shows the “uncrossed” disparity in which the wedges were farther from the observer than the fixation plane (i.e., disparity far). The magnitude of the disparity was the same (i.e., disparity near = - disparity far). (C) Diagram of the perceived depth arrangement in the stimuli. Four disparity-defined wedges were simultaneously presented at near or far disparity. For the correlated stimuli, participants perceived the wedge structure; note that the black outline on the wedges is for illustrative purposes only. For the anti-correlated stimuli, the position of the wedges is indicated by dashed outlines; however, participants could not perceive the wedge structure. (D) Example stimuli used in the experiment, designed for red-cyan anaglyph viewing. Vernier task stimuli were presented on the centre of the stimulus as shown by the lines of the cross hair.

Here, we employ sub-millimetre fMRI to investigate the contribution of feedforward and feedback mechanisms in visual cortex related to 3D perception. We compare fMRI responses across cortical depths to stimuli that enable stable stereoscopic perception (i.e., correlated random dot stereograms: RDS) vs. anti-correlated RDS stimuli that do not support a unified perceptual experience of 3D structure (Fig. 2A). Using multi-voxel pattern analysis (MVPA) across cortical depths, we demonstrate cortical depth-specific representations in area V3A and V7; that is, multivoxel pattern responses were stronger for correlated than anti-correlated stimuli in upper than deeper layers. Further, we show higher feedforward connectivity for correlated than anti-correlated stimuli between superficial layers of V3A and middle layers of V7, suggesting that V3A propagates 3D structure information to higher dorsal areas. Finally, we show higher feedback connectivity for correlated than anti-correlated stimuli between deeper layers of a) V3A and V1, b) V7 and V3A, suggesting top-down influences on binocular disparity processing. Our findings indicate that area V3A is a key nexus for disparity processing that gates feedforward and feedback interactions to support 3D perception.

## Materials and Methods

### Participants

Eight healthy volunteers (6 females; age range: 22–31 years) participated in the study. The sample size followed previous 7T fMRI studies (e.g., Goncalves et al., 2015; N = 6) on binocular disparity using RDS stimuli. Data from one participant were excluded from further analysis due to excessive head movement (higher than 1.5 mm). All participants had normal or corrected-to-normal vision and were screened for stereo deficits prior to entering the scanner. Participants gave written informed consent and received payment for their participation. The study was approved by the local Ethical Committee of the Faculty of Psychology and Neuroscience at Maastricht University.

### Stimuli

The stimuli were random dot stereogram images consisting of black and white dots on a mid-grey background. Within these patterns we imposed a disparity structure that defined four ‘wedges’ that were either in front or behind the background plane of fixation (Fig. 2B). These wedges were only visible once binocular correspondence had been established (Fig. 2C–D): if viewing the stimuli monocularly only randomly placed dots were apparent within a square region containing the stimulus (side length: 8°). The wedges were presented centrally around a circular aperture (diameter: 1.2°) containing the fixation marker that was positioned in the plane of the screen (Goncalves et al., 2015). Each wedge subtended 7° in the radial direction and 70° in polar angle. To reduce adaptation effects, we changed the position of the disparity-defined edges of the wedge stimulus across stimulus presentations by randomly rotating the wedge regions clockwise or counterclockwise. The stimuli (wedges and the background) were presented in correlated or anti-correlated form of dense RDS (Preston et al., 2008). For the correlated RDS patterns (Fig. 2A), the polarity of the dots presented matched between eyes (e.g., a black dot in the left eye matched a black dot in the right eye, while a white dot in the left eye matched a white dot in the right eye). In the anti-correlated RDS (Fig. 2A), we inverted the contrast polarity between the eyes (e.g., a black dot in the left eye matched a white dot in the right eye, and a white dot in the left eye matched a black dot in the right eye). When participants viewed correlated stimuli, they were able to perceive the wedges in front or behind the background. When participants viewed the anti-correlated stimuli, the whole stimulus appeared lustrous and there was no consistent impression of 3D structure. The background and fixation target were presented at the plane of the screen (i.e., zero disparity). The wedges were presented at near or far perceived depth corresponding to crossed vs. uncrossed disparities (±10 arcmin with ±0.5 arcmin jitter, Fig. 2B). In the “crossed” disparity configuration, the stimulus was closer (near) to the observer than the fixation plane; in “uncrossed” disparity configuration, the stimulus was farther (far) from the observer than the fixation plane. The four wedges were simultaneously rendered with the same binocular correlation form and disparity. Further, a static grid with squares surrounded the RDS (not depicted in Figure 2), providing an unambiguous reference and enabled stable vergence during fixation.

To avoid confusion between the perception of 3D structure (i.e., ‘depth’ perception) and different sampling positions relative to the cortical surface (i.e., cortical ‘depth’), within this paper we reserve the term ‘depth’ to refer to neuroanatomical positions. We refer to the perception of 3D structure as 3D/stereoscopic perception.

Experiments were controlled using MATLAB (The MathWorks, Inc., Natick, MA, USA) and Psychophysics toolbox 3.0 (Brainard, 1997; Pelli, 1997). Stimuli were presented using a projector and a mirror setup (1920 × 1080 pixels resolution, 60 Hz frame rate) at a viewing distance of 99 cm. Participants viewed red and cyan anaglyphs through colour-filter glasses.

## Experimental design and statistical analysis

### fMRI design

A 2 × 2 block experimental design was used with factors Stereo (correlated vs. anti-correlated RDS) and Disparity (near vs. far). The fMRI experiment comprised a maximum of 6 runs (3 participants completed 6 runs; 1 participant completed 5 runs; 2 participants completed 4 runs; 1 participant completed two scans of 6 runs and 4 runs in two separated day). The amount of time available to collect experimental data depended on the initial set up time for each participant (e.g., testing visibility of the RDS stimuli in the scanner, MRI-related calibrations (e.g., shimming)).

Each run lasted 8 min 24 s, started with a fixation block (12 s), followed by 40 stimulus blocks, ten blocks for each of the four conditions, and ended with a fixation block (12 s). The order of the blocks was counterbalanced within and across runs and participants. Each block lasted 12 s and comprised ten stimuli of the same condition. Each stimulus was displayed for 900 ms followed by a 300 ms inter-stimulus interval, with a randomized jitter of disparity (±0.5 arcmin).

During scanning, participants engaged in an attentionally demanding dichoptic Vernier detection task at the fixation marker (for details, see Popple et al., 1998; Murphy et al., 2013). Briefly, participants were instructed to fixate a central cross hair fixation marker that was present throughout experimental scans. Two of the lines of the cross hair were presented to one of the eyes (e.g., top and left lines in the left eye) and the other two to the other eye (e.g., bottom and right lines in the right eye) (see Fig. 2D). Participants were instructed to alter their horizontal and/or vertical eye vergence so that the cross hairs were properly aligned to form a cross. In addition, a small additional Vernier target was briefly-flashed (900 ms) to one eye, and the participants were asked to indicate its position (left vs. right under a two-alternative forced choice paradigm) relative to the top line of the fixation marker.

To localize the visual areas, a region of interest (ROI) localizer scan was also acquired. A circular chequerboard of the size of the RDS stimuli was presented flickering at 8 Hz. The localizer scan lasted 5 min 36 s, and started with a 16 s fixation, followed by 20 stimuli. Each stimulus was displayed for 2 s with a 14 s inter-stimulus interval. In the same scanning session, anatomical data and fMRI data for retinotopic mapping were collected following standard procedures (e.g., Engel et al., 1997).

### MRI data acquisition

Imaging data were acquired on a 7T Magnetom scanner (Siemens Medical System, Erlangen, Germany) at the Scannexus Imaging Centre, Maastricht, The Netherlands. We used a 32-channel phased-array head coil (NOVA Medical, Wilmington, MA, USA) and a 2D Gradient Echo-Echo Planar Imaging (GE-EPI) sequence (Moeller et al., 2010; TR = 2 s, TE = 25 ms, voxel size = 0.8 mm isotropic, FOV = 148 × 148 mm^2^, number of slices = 56, partial Fourier = 6/8, GRAPPA factor = 3, Multi-Band factor = 2, bandwidth = 1168 Hz/Pixel, echo spacing = 1 ms, flip angle = 70°). The FOV covered occipito-temporal and posterior parietal areas; manual shimming was performed prior to the acquisition of the functional scans. Anatomical images were acquired using MP2RAGE T1-weighted sequence (TR = 5 s, TE = 2.51 ms, voxel size = 0.65 mm isotropic, FOV = 208 × 208 mm^2^, 240 sagittal slices).

### MRI data analysis

#### Anatomical data analyses

**Figure 3:**
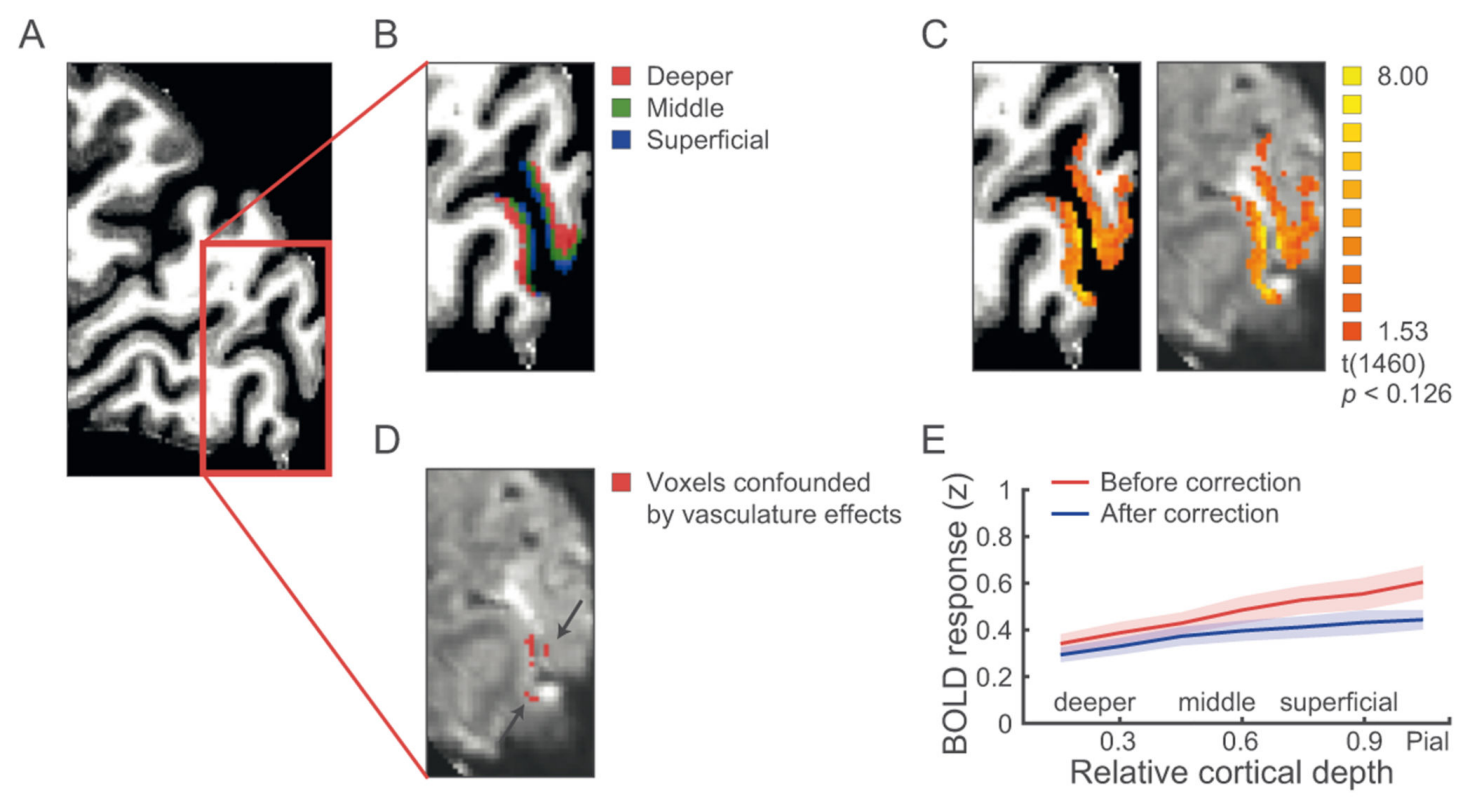
fMRI layer definition and vascular correction. (A) Sagittal (top panel) and transverse (bottom panel) view of the anatomical image of a sample participant. Red inserts indicate ROIs in visual cortex. (B) Layers definition map overlaid on an anatomical image (Top panel: V1: red, deeper layers; green, middle layers; blue, superficial layers; Bottom panel: V3A: red, deeper layers; green, middle layers; blue, superficial layers; V7: purple, deeper layers; light green, middle layers; light blue, superficial layers). V7 definition followed the Wang et al. (2015) atlas and was validated against the atlas derived from the HCP data (Benson et al., 2018) showing 83% overlap in ROI definition. MVPA analysis showed similar results across atlases used for ROI definition (significant cortical depth (deeper, middle, superficial layers) × condition (correlated, anti-correlated RDS) interaction for MVPA accuracy in V7 using the Wang et al. atlas (F(2,12) = 4.37, *p* = 0.037) or the HCP atlas (F(2,12) = 4.74, *p* = 0.030). (C) BOLD activation map (stimulus vs. fixation) overlaid on the anatomical (left panel) and functional data (right panel) in V1. (D) Voxels confounded by vasculature-related effects (red, highlighted by arrows) overlaid on mean functional image in V1. (E) Mean normalized BOLD in V3A before (red) and after correction (blue) for vasculature-related effects across cortical depth, showing reduced superficial bias after correction. Similar results were observed for V1 and V7. The stronger BOLD decrease in upper (i.e., superficial, middle) than deeper layers after correction suggests that our approach for correcting vasculature-related effects controlled substantially for the superficial bias. Shaded areas indicate SEM across participants.

T1-weighted anatomical data was used for coregistration and 3D cortex reconstruction. Grey and white matter segmentation (Fig. 3A) was obtained on the MP2RAGE images using FreeSurfer (https://surfer.nmr.mgh.harvard.edu; Fischl, 2012) and manually improved for the ROIs (i.e., V1, V3A, V7) using ITK-SNAP (https://www.itksnap.org; Yushkevich et al., 2006). The refined segmentation was used to obtain a measurement of cortical thickness. Following previous studies, we assigned voxels to three cortical depths (deeper, middle, superficial layers; Fig. 3B) using the equi-volume approach (Waehnert et al., 2014; Kemper et al., 2018) as implemented in BrainVoyager (version 20.6; Brain Innovation, Maastricht, The Netherlands). This approach has been shown to reduce misclassification of voxels to layers, in particular for ROIs presenting high curvature. Information from the cortical thickness map and gradient curvature was used to generate four grids at different cortical depths (ranging from 0: white matter, to 1: grey matter). Mapping of each voxel to a layer was obtained by computing the Euclidean distance of each grey matter voxel to the grids: the two closest grids represent the borders of the layer to which a voxel is assigned (Fig. 3C). Note that due to limitations in the UHF imaging resolution these MRI-defined layers indicate distance (i.e., cortical depth) from the grey matter/white matter and the grey matter/cerebrospinal fluid boundaries rather than one-to-one mapping to the cyto-architectonically defined layers of the human neocortex.

#### fMRl data analyses

The functional data were analysed using BrainVoyager and custom MATLAB code. The first volume at the beginning of each run was discarded to ensure that longitudinal magnetization reached steady state. Preprocessing of the functional data involved three steps starting with correction of distortions due to non-zero off-resonance field; that is, at the beginning of each functional run, five volumes with inverted phase encoding direction were acquired and used to estimate a voxel displacement map that was subsequently applied to the functional data using the COPE (Correction based on Opposite Phase Encoding, BrainVoyager, Brain Innovation). The distortion-corrected data underwent slice-timing correction, head motion correction (the single band image acquired at the beginning of each run was used as reference in the alignment), high-pass temporal filtering (using a GLM with Fourier basis set at 2 cycles), and removal of linear trends. To validate the alignment, we calculated the mean EPI image of each functional run for each ROI and estimated the spatial correlation between these mean EPI images. We performed manual adjustment of the alignment if the spatial correlation was below 0.85 (Marquardt et al., 2018). Preprocessed functional data were coaligned to the anatomical data using the boundary-based registration approach, as implemented in BrainVoyager (Greve and Fischl, 2009). Results were manually inspected and further adjusted where needed.

#### ROIs definition

We used the data from the retinotopic mapping scan to identify visual areas V1 and V3A based on standard phase-encoding methods. Participants viewed rotating wedges that created traveling waves of neural activity (e.g., Sereno et al., 1995; Engel et al., 1997). Area V7 was defined at the posterior intra-parietal sulcus for each participant based on anatomical templates following Wang et al. (2015) provided by Benson (https://hub.docker.com/r/nben/occipital_atlas/; Benson et al., 2012; Benson et al., 2014). This procedure uses the individual participant-based segmentation obtained with FreeSurfer and an anatomical probabilistic template, to estimate the best location for the ROI (i.e., V7). Our definition of V7 was consistent with the atlas derived from Human Connectome Project (Benson et al., 2018). We found an 83% overlap in the definition of V7 between atlases and the results showed similar patterns when comparing across ROI definitions. Each area was subsequently inspected to ensure consistent definition across participants; that is, we checked whether the ROI: (1) was located at the correct anatomical location; and (2) covered only grey matter, rather than white matter or CSF.

For each ROI and participant, we modelled BOLD signals using a GLM with four regressors, one per stimulus condition (stereo × disparity), and included the estimated head motion parameters as nuisance regressors. Similarly, we modelled the localizer BOLD signals using a GLM with one stimulus regressor, fixation, and motion parameters as nuisance regressors. The resulting t-statistical map was thresholded (main scan: *t* = 1.53, *p* = 0.125; localizer scan: *t* = 1.96, *p* = 0.05) to select voxels within each ROI that showed stronger responses to the stimulus conditions compared to fixation baseline (Fig. 3C).

#### Correcting for vasculature-related effects

Voxel selection within each ROI was further refined by excluding voxels that were confounded by vasculature effects that are known to contribute to a superficial bias in the measured BOLD signal; that is, increased BOLD with increasing distance from white matter. In particular, it has been shown that the BOLD signal measured using GE-EPI (i.e., T2*-weighted) is confounded by macro- and micro-vasculature signals (Uğurbil et al., 2003; Yacoub et al., 2005; Uludağ et al., 2009). The macro-vasculature contribution is due to veins penetrating the grey matter and running through its thickness, as well as large pial veins situated along the surface of the grey matter (Duvernoy et al., 1981). This results in increased sensitivity (i.e., strong BOLD effect) but decreased spatial specificity of the measured signal. The latter can be understood by the mechanics of the draining veins carrying deoxygenated haemoglobin downstream from the true neuronal site of neural activation, leading to a response spatially biased towards the pial surface, an effect known as superficial bias.

Here, we took the following approach to reduce superficial bias due to vasculature contributions (Jia et al., 2020; Zamboni et al., 2020). First, following previous work (Olman et al., 2007), we computed the temporal signal to noise ratio (tSNR) for each voxel in each ROI (V1, V3A, V7). We used tSNR to identify voxels near large veins that are expected to have large variance and low intensity signal due to the local concentration of deoxygenated haemoglobin resulting in a short T2* decay time (i.e., dark intensity in a T2*-weighted image). We identified voxels with low tSNR (lower than 2 SD; mean tSNR across V1 smaller than 13.26 ± 1.02, V3A: smaller than 14.46 ± 1.31, V7 smaller than 13.99 ± 1.75) and checked their correspondence with voxels of lower intensities on the T2*-weighted images. Second, it has been shown that high t-values on an fMRI statistical map are likely to arise from large pial veins (Polimeni et al., 2010; Kashyap et al., 2018). Therefore, voxels with low tSNR values or *t*-score values above the 90^th^ percentile (mean *t*-score across V1 larger than 6.24 ± 1.76, V3A larger than 3.84 ± 1.00, V7 larger than 3.11 ± 0.48) of the *t*-score distribution obtained by the GLM described above were removed from further analysis. We used these two approaches to correct the BOLD signal from confounding vasculature effects.

Further to account for possible differences in signal strength across cortical layers due to thermal and physiological noise, as well as signal gain (Goense et al., 2012; Havlicek and Uludağ, 2020), we: (1) matched the number of voxels across layers per participant and ROI; and (2) z-scored the time courses within cortical layer per ROI, controlling for differences in signal levels across layers while preserving signal differences across conditions (after correction of vascular contributions, e.g., Lawrence et al., 2019a). To validate this approach, we compared the mean normalized fMRI responses before and after correction. For each participant and run, we extracted the mean normalized fMRI responses per block. To account for hemodynamic delay in our blocked design, we shifted the time course by 2 TRs (i.e., 4 s) from the onset of the block. Further to capture the BOLD signal from the last stimulus presentation per block we included signals from 1 TR after the end of each block. That is, we included fMRI responses between the 3^rd^ and 7^th^ TR (i.e., 4–14 s) after stimulus onset. The normalized fMRI responses were averaged across time points, stimulus presentation, and runs. Repeated-measures ANOVA was used to test the BOLD signal (before vs. after correction) and cortical depths (deeper, middle, superficial layers) (see Results, Control analyses).

#### Multivariate pattern analysis

Following our previous work (Preston et al., 2008; Jia et al., 2020), we used MVPA to discriminate BOLD responses related to near vs. far disparity stimuli across cortical layers in visual cortex. For each ROI and participant, we calculated per voxel a *t*-score statistic by comparing activity for stimuli vs. fixation. We used this statistic to rank the voxels within cortical depths (deeper, middle, superficial layers) per ROI and selected 175 voxels per layer with the higher t-score to include in the MVPA, as MVPA accuracy saturated across all participants for these voxel pattern sizes in the corresponding regions. This voxel selection procedure ensured that comparisons of MVPA accuracy could not be confounded by varying number of voxels across participants. We then extracted mean normalized fMRI responses between 3^rd^ to 7^th^ TR (i.e., 4–14 s) after block onset for this pattern of voxels per ROI and participant. We trained a linear classifier using LIBSVM with default C value (C = 1) (https://www.csie.ntu.edu.tw/~cjlin/libsvm/, Chang and Lin, 2011) implemented in MATLAB to discriminate the near from the far disparity stimulus. We computed MVPA accuracy using a leave-one-run-out cross-validation. That is, we divided the data set into training (60–180 patterns depending on the number of scanning runs per participant) and test (20 patterns) data. We averaged the MVPA accuracy across folds. We used repeated-measures ANOVAs to assess differences in MVPA accuracy across conditions (correlated, anti-correlated RDS), cortical depths (deeper, middle, superficial layers), and ROIs (V1, V3A, V7). Mauchly’s W tests were used to determine if the assumption of sphericity was violated. If necessary, we corrected the degrees of freedom by the Greenhouse-Geisser correction (for ε < 0.75). Post-hoc comparisons were carried out using pairwise t-test comparisons. Within-subject SEM was adjusted using the Cousineau-Morey method (O’Brien and Cousineau, 2014).

To corroborate the MVPA analysis we conducted a cross-validated linear discriminant contrast (LDC) analysis (Diedrichsen et al., 2016; Walther et al., 2016). The linear discriminant contrast is centred on zero under the null hypothesis of no reliable differences between the near vs. far conditions. We used the same data and voxels as in the MVPA analysis. We divided the data set into training and test data and performed a leave-one-run-out cross-validation. For each cross validation, we contrasted signals from the near against the far stimulus blocks to generate the representation distance metric for both the training and test datasets. The distance matrix from the training datasets was normalized using the sparse covariance matrix of the noise residuals to produce the weights vector. The LDC is the dot product of the representation distance metric for the test dataset and the weight matrix estimated from the training dataset. Finally, we averaged the LDC values across cross validations per participant.

#### Informational connectivity analysis

We used informational connectivity (IC) to identify layers that share synchronized discriminability of activity related to stimulus-specific multi-voxel pattern information (Coutanche and Thompson-Schill, 2014; Anzellotti and Coutanche, 2018; Koster et al., 2018; Jia et al., 2020). We examined intercortical informational connectivity based on shared changes (fluctuations) in pattern discriminability over time, as this approach has been shown to be more sensitive than univariate functional connectivity. To track the flow of multivariate information across time (i.e., across blocks), we measured the fluctuations (covariance) in MVPA discriminability by calculating distance information from the classification hyperplane. In particular, we selected 175 voxels with the higher *t*-score and used the same multivoxel near vs. far disparity patterns as in the MVPA analysis. For each ROI and layer, we extracted distance information for the test data per block from the trained classifiers. We calculated layer-specific connectivity by Spearman correlation between the fold-wise distance of different layers. We transformed the correlation coefficients using Fisher’s z-transform and conducted repeated-measures ANOVAs to compare across conditions (correlated, anti-correlated RDS) and ROI pairs (V1-V3A, V3A-V7, V1-V7) per pathway (feedforward, feedback). Note that both feedforward and feedback processing involve interactions between superficial layers of lower visual areas and deeper layers of higher visual areas; that is, we cannot differentiate feedforward from feedback processing based on superficial-to-deeper layer connectivity. Thus, we focused our investigations on the connectivity between deeper layers that is known to be related to feedback processing (Rockland and Pandya, 1979; Maunsell and van Essen, 1983).

## Results

### Disparity representations across cortical depths

We tested fMRI responses across cortical depths in the visual cortex when participants were presented with disparity-defined stimuli. We rendered binocular disparities in random dot stereograms (RDS) in which the 3D structure of the stimulus is only apparent once binocular correspondence has been established. Correlated RDS stimuli (cRDS, Fig. 2A) support a clear interpretation of stereoscopic structure, however the same disparity information can be rendered in anti-correlated RDS stimuli (aRDS, Fig. 2A) that do not give rise to a clear perceptual interpretation of depth.

These stimuli are informative because while dense aRDS do not support a perceptual interpretation of 3D structure, the disparities they contain are registered by neurons in the primary visual cortex (Cumming and Parker, 1997), but not at higher levels of the visual system (e.g., Janssen et al., 2003). Anti-correlated stimuli have therefore been interpreted as ‘false matches’ that should be vetoed to solve the stereo “correspondence problem” (Scharstein and Szeliski, 2002) and produce a close similarity between neural responses and the perceptual interpretation of 3D structure. Thus, contrasting fMRI signals for correlated vs. anti-correlated stimuli provides a useful means to test neural representations that relate to stereoscopic perception. We rendered all stimuli (correlated, anti-correlated) to contain either crossed or uncrossed binocular disparities (nearer or further from the fixation point; Fig. 2B). We compared fMRI responses for these stimuli (crossed vs. uncrossed) across cortical depths in areas V3A and V7 that are known to be engaged by correlated RDS stimuli (Preston et al., 2008; Goncalves et al., 2015) vs. V1 that is known to respond to both correlated and anti-correlated stimuli (Bridge and Parker, 2007; Preston et al., 2008; Ip et al., 2014).

For each participant, we segmented the visual areas and assigned voxels to three cortical depths (deeper, middle, superficial layers) using an equi-volume approach (see Material and Methods, MRI data analysis: Anatomical data analyses; Fig. 3A–B). Voxels identified as containing large veins were removed from the analysis, to improve the spatial specificity of the cortical depth profiles (Fig. 3C–D). We used MVPA to discern disparity-specific (i.e., near vs. far disparities) fMRI signals when participants were presented with correlated vs. anti-correlated RDS stimuli. In particular, we tested whether linear classifiers that were trained on fMRI signals from multi-voxel patterns across cortical depths (deeper, middle, superficial) in V1, V3A, and V7 discriminated between near vs. far disparity stimuli based on differences in crossed vs. uncrossed disparity signals. We hypothesised that higher MVPA accuracy for correlated compared to anti-correlated RDS stimuli would indicate disparity-specific representations related to stereoscopic perception.

**Figure 4:**
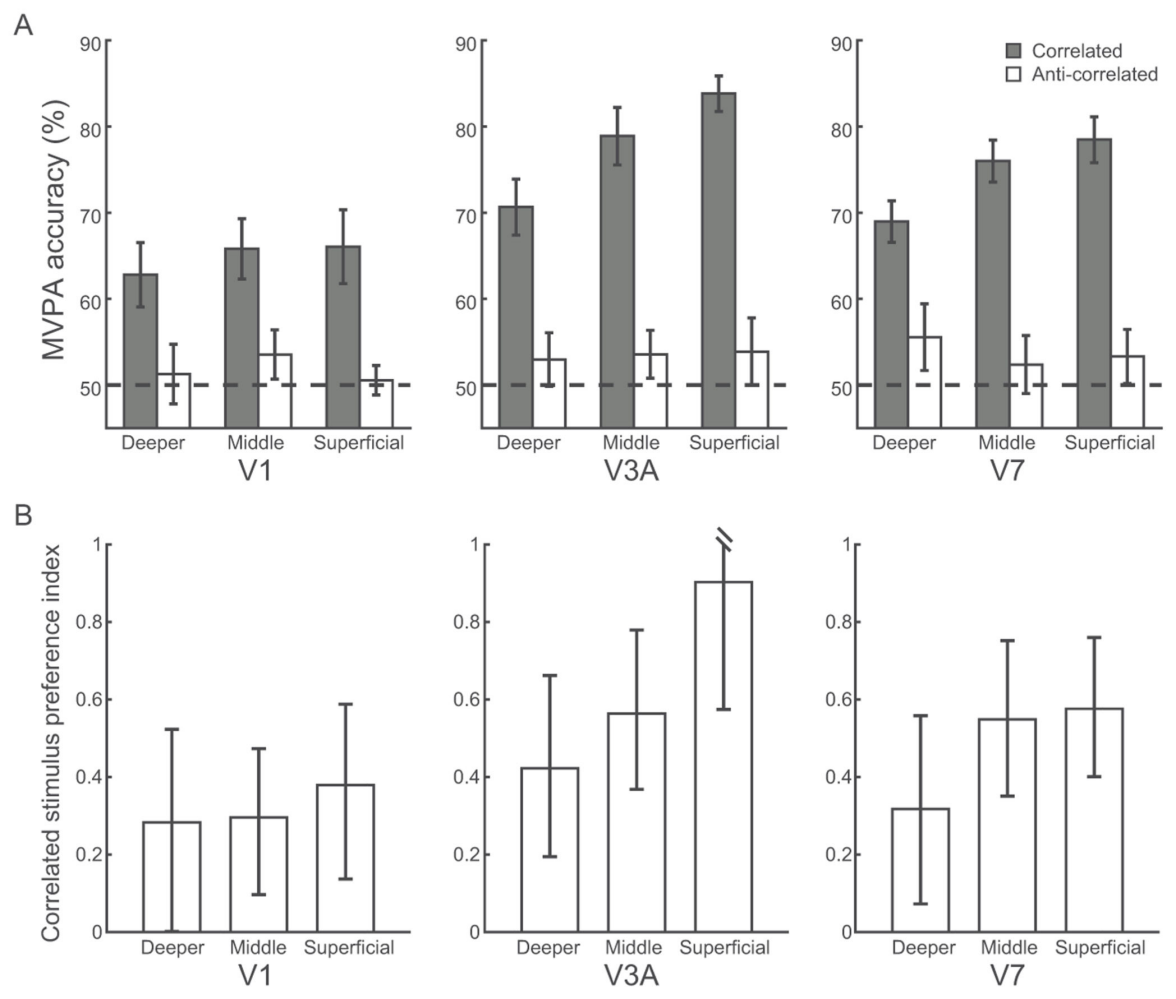
Pattern classification analyses across cortical depth. (A) MVPA accuracy across V1, V3A, V7 layers for correlated (grey bar) and anti-correlated (white bar) stimuli in the near vs. far disparity classification. Dotted line indicates MVPA accuracy at 50% chance. (B) Linear discriminant contrast distance across V1, V3A, V7 layers for correlated (grey bar) and anti-correlated (white bar) stimuli in the near vs. far disparity contrast. Error bars indicate within-subject SEM across participants. Filled dots indicate individual data.

A three-way repeated-measures ANOVA (condition: correlated, anti-correlated RDS; cortical depth: deeper, middle, superficial layers; ROI: V1, V3A, V7) showed significant main effects of condition (correlated, anti-correlated RDS; F(1,6) = 30.42, *p* = 0.001, Fig 4A) and cortical depth (deeper, middle, superficial layers; F(2,12) = 4.286, *p* = 0.039), but no significant main effect of ROI (V1, V3A, V7; F(1.2,7.4) = 3.60, *p* = 0.093). We observed a significant condition × cortical depth interaction (F(2,12) = 8.65, *p* = 0.005) and a significant condition × ROI interaction (F(2,12) = 7.08, *p* = 0.009), but no significant 3-way interaction (F(4,24) = 1.15, *p* = 0.358).

Further, a two-way repeated-measures ANOVAs (condition × cortical depth) in V3A showed significantly higher MVPA accuracy for upper (i.e., superficial, middle) than deeper layers (main effect of cortical depth: F(2,12) = 7.01, *p* = 0.010), for correlated than anti-correlated stimuli (main effect of condition: F(1,6) = 38.03, *p* = 0.001), and a significant condition × cortical depth interaction (F(2,12) = 6.02, *p* = 0.015), suggesting layer-specific disparity processing in V3A. Post-hoc comparisons showed that MVPA accuracy for correlated stimuli was significantly higher in superficial (t(6) = 3.13, *p* = 0.020) and middle (t(6) = 2.45, *p* = 0.049) than deeper layers. There were no significant differences between superficial and middle layers for correlated stimuli (t(6) = 2.26, *p* = 0.065).

Similarly, for V7 a two-way repeated-measures ANOVA (condition × cortical depth) showed significantly higher MVPA accuracy for upper than deeper layers (main effect of cortical depth: F(2,12) = 4.37, *p* = 0.037), for correlated than anti-correlated stimuli (main effect of condition: F(1,6) = 31.46, *p* = 0.001), and a significant condition × cortical depth interaction (F(2,12) = 4.97, *p* = 0.027), suggesting layer-specific disparity processing in V7. Post-hoc comparisons showed that MVPA accuracy for correlated stimuli was higher in superficial (t(6) = 3.85, *p* = 0.008) and middle (t(6) = 2.62, *p* = 0.040) than deeper layers. There were no significant differences between superficial and middle layers for correlated stimuli (t(6) = 0.33, *p* = 0.751).

In contrast to the MVPA results in V3A and V7, in V1 a two-way repeated-measures ANOVA (condition × cortical depth) showed significantly higher MVPA accuracy for correlated than anti-correlated stimuli (main effect of condition: F(1,6) = 10.24, *p* = 0.019), but no significant main effect of cortical depth (F(2,12) = 0.90, *p* = 0.431) nor condition × cortical depth interaction (F(1.1,6.3) = 1.41, *p* = 0.280).

Finally, a cross-validated linear discriminant contrast (LDC) analysis corroborated our results on MVPA accuracy (Diedrichsen et al., 2016; Walther et al., 2016). A three-way repeated-measures ANOVA showed a significant three-way interaction (condition × cortical depth × ROI, F(4,24) = 6.35, *p* = 0.001) (Fig. 4B). Further, two-way repeated-measures ANOVAs on the LDC distance per ROI showed a significant two-way interaction (condition × cortical depth) in V3A (F(1.1,6.9) = 15.21, *p* = 0.005) and V7 (F(2,12) = 12.06, *p* = 0.001), but not in V1 (F(2,12) = 2.92, *p* = 0.092). Post-hoc comparisons in V3A showed higher LDC distance for correlated stimuli in superficial than deeper (t(6) = 4.16, *p* = 0.018) and middle (t(6) = 4.85, *p* = 0.009) layers, but not middle vs. deeper layers (t(6) = 3.21, *p* = 0.055). Post-hoc comparisons in V7 showed higher LDC distance for correlated stimuli in superficial vs. deeper layers (t(6) = 4.78, *p* = 0.009), but not superficial vs. middle layers (t(6) = 2.67, *p* = 0.111) or middle vs. deeper layers (t(6) = 2.96, *p* = 0.076).

Taken together our results suggest that disparity representations for stereoscopic perception (i.e., higher classification accuracy for correlated than anti-correlated stimuli) in V3A and V7 are layer-specific. Despite the lack of a significant three-way interaction on MVPA accuracy (condition × cortical depth × ROI), potentially due to the small sample size, the LDC distance analysis showed a significant condition × cortical depth × ROI interaction. This analysis suggests disparity representations for stereoscopic perception in V3A and V7 that are specific to upper rather than deeper cortical layers. Finally, the differences in MVPA accuracy between correlated and anti-correlated stimuli could not be simply due to differences in attention, as participants performed an attentionally demanding task on the fixation point during scanning. Further, our stimuli were designed to reduce differences in vergence eye movements across conditions. In particular, (1) a stable, low spatial frequency pattern in the plane of the screen surrounded the stimuli; and (2) participants were instructed to use the horizontal and vertical nonius lines to assist them in ensuring correct eye alignment at all times.

### Control analyses

To validate our results and control for potential confounds we conducted the following additional analyses.

First, it has been shown that overall BOLD signal as measured by GE-EPI is higher at the cortical surface due to vascular contributions (Uğurbil et al., 2003; Yacoub et al., 2005; Uludağ et al., 2009) resulting in loss of spatial specificity (Kay et al., 2019). Following our previous work (Jia et al., 2020; Zamboni et al., 2020), we combined several approaches to reduce this superficial bias by removing voxels with low temporal signal-to-noise ratio and high t-statistic for stimulation contrast (see Material and Methods, MRI data analysis: Correcting for vasculature-related effects for details). We then z-scored each voxel’s time course to account for possible differences in signal strength and variance due to thermal or physiological noise across layers while preserving differences between conditions (Lawrence et al., 2019a). Figure 3E shows that following these corrections the superficial bias was significantly reduced in V3A. That is, the magnitude of BOLD signals from voxels closer to the pial surface were reduced, as indicated by a significant interaction between BOLD signal from different cortical depths (deeper, middle, superficial layers) before vs. after correction (F(2,12) = 28.61, *p* < 0.001). That is, the superficial bias corrections resulted in decreased BOLD signal in upper layers as indicated by post-hoc comparisons (middle layers: t(6) = 6.95, *p* < 0.001; superficial layers: t(6) = 7.56, *p* < 0.001). Similar reduction in superficial bias was observed in V1 (F(1.1,6.6) = 18.89, *p* = 0.003; post-hoc comparisons: middle layers: t(6) = 12.57, *p* < 0.001; superficial layers: t(6) = 12.07, *p* < 0.001) and V7 (F(1.1,6.3) = 16.25, *p* = 0.006; post-hoc comparisons: middle layers: t(6) = 5.24, *p* = 0.002; superficial layers: t(6) = 4.88, *p* = 0.003). Thus, it is unlikely that our MVPA results after vasculature correction were significantly confounded by the superficial bias.

**Figure 5:**
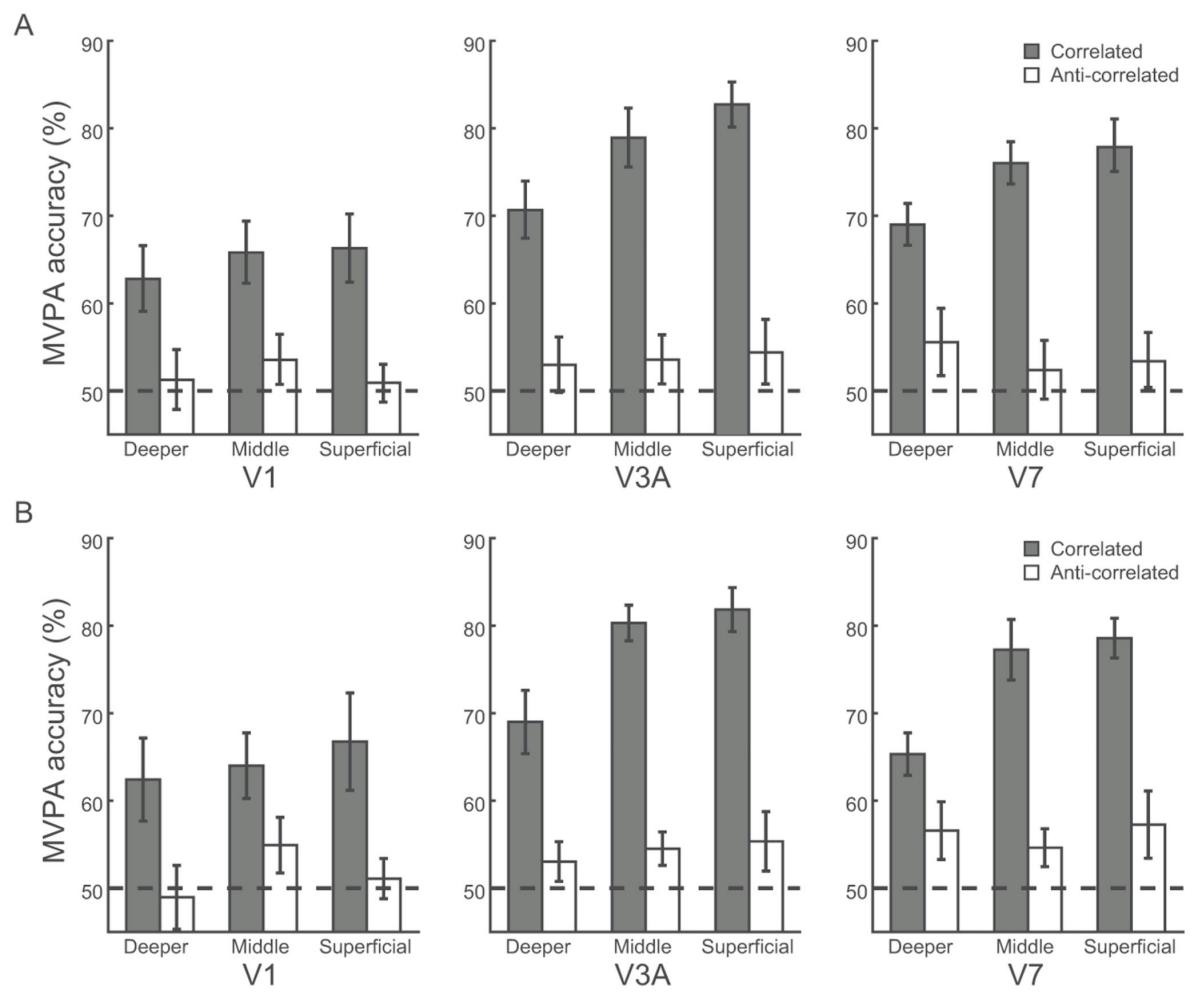
Control analyses. (A) MVPA accuracy for correlated (grey bar) and anti-correlated (white bar) stimuli in the near vs. far disparity classification in V1, V3A, V7 layers after regressing out the signal from the adjacent voxels in middle layers. (B) MVPA accuracy for correlated (grey bar) and anti-correlated (white bar) stimuli in the near vs. far disparity classification in V1, V3A, V7 layers after regressing out mean voxel response per condition (i.e., univariate information). Dotted line indicates MVPA accuracy at 50% chance. Error bars indicate within-subject SEM across participants. Filled dots indicate individual data.

Second, we applied a spatial regression approach (Kok et al., 2016; Markuerkiaga et al., 2016; Koster et al., 2018) to control for signal contribution from draining veins. In particular, intra-cortical veins running perpendicular to the cortical surface are known to drain blood from deeper layers of the cortex to larger pial veins situated along the grey matter surface, resulting in loss of spatial specificity and intra-layer BOLD signal contamination. To unmix the signal from adjacent layers, for each voxel in the superficial layers, we found its nearest neighbours in the middle layers. We then averaged the time course of these voxels and regressed the mean time course out of each voxel in the superficial layers. MVPA analysis following this correction showed a significant condition × cortical depth interaction in V3A (F(2,12) = 7.27, *p* = 0.009, Fig. 5A) and V7 (F(2,12) = 4.73, *p* = 0.031). Further, we observed higher MVPA accuracy for correlated stimuli in superficial (t(6) = 2.45, *p* = 0.049) and middle (t(6) = 2.91, *p* = 0.027) than deeper V3A layers, but not superficial vs. middle layers (t(6) = 2.30, *p* = 0.061); and higher MVPA accuracy for correlated stimuli in superficial (t(6) = 3.85, *p* = 0.008) and middle (t(6) = 2.68, *p* = 0.037) than deeper V7 layers, but not superficial vs. middle layers (t(6) = 0.39, *p* = 0.713), consistent with stronger stereoscopic processing in upper than deeper V3A and V7 layers. That is, our results remained significant after these corrections, suggesting that our results are unlikely to be significantly confounded by vasculature-related artifacts.

Third, to ensure that our classification approach was not overpowered and did not suffer from any bias, we ran the classification with the data labels shuffled. Theoretically, this should result in classification accuracies at chance. The results for the classification of 5000 permutations of shuffled data for the near vs. far classification did not differ significantly from chance (all *ps* > 0.500, false discovery rate corrected), suggesting that our MVPA analysis extracted reliable voxel pattern information.

Fourth, to ensure that differences in univariate BOLD across ROIs and cortical depths did not account for our MVPA results, we trained the classifier after regressing out the mean normalized response across voxels for each ROI and condition. This analysis showed similar results (Fig. 5B) to the main MVPA (Fig. 4A): two-way repeated-measures ANOVAs showed significant condition × cortical depth interaction in V3A (F(2,12) = 9.87, *p* = 0.003) and V7 (F(2,12) = 4.82, *p* = 0.029), but not in V1 (F(1.1,6.4) = 0.30, p = 0.617), suggesting that our MVPA results relate to multivoxel pattern representations of 3D structure rather than overall BOLD signal in each ROI.

Fifth, to ensure that our results were not specific to voxel number (n = 175) selected for MVPA, we tested the MVPA analysis with different voxel pattern sizes (i.e., 200, 225, and 250 voxels). The MVPA analyses using these voxel patterns showed similar results. In particular, for 200 voxels, two-way repeated-measures ANOVAs showed significant condition × cortical depth interaction in V3A (F(1.1,6.5) = 6.46, p = 0.039) and V7 (F(2,12) = 7.19, p = 0.009), but not in V1 (F(2,12) = 0.18, p = 0.841). For 225 voxels, two-way repeated-measures ANOVAs showed significant condition × cortical depth interaction in V3A (F(2,12) = 4.00, p = 0.047) and V7 (F(2,12) = 7.48, *p* = 0.008), but not in V1 (F(2,12) = 0.28, p = 0.762). For 250 voxels, two-way repeated-measures ANOVAs showed significant condition × cortical depth interaction in V3A (F(2,12) = 4.12, p = 0.043) and V7 (F(2,12) = 5.17, p = 0.024), but not in V1 (F(2,12) = 0.31, p = 0.738). These results are consistent with our main MVPA analysis using 175 voxels, suggesting that our results are unlikely to be significantly affected by choice of pattern size.

### Informational connectivity analysis

UHF fMRI allows us to interrogate the finer functional connectivity across brain areas based on known anatomical models of connectivity across cortical layers. Here, we tested functional connectivity across visual areas (V1, V3A, V7) to investigate the role of feedforward and feedback processing for stereoscopic perception. While UHF imaging resolution does not support one-to-one mapping between MRI-defined cortical depths and cyto-architectonically defined layers, previous UHF imaging studies (e.g., Kok et al., 2016; Huber et al., 2017; Sharoh et al., 2019; Moerel et al., 2020) have provided a framework of feedback and feedforward connections across deeper, middle, and superficial cortical depths (Fig. 1).

Using this framework, we computed functional connectivity between V3A, earlier (i.e., V1) and higher (i.e., V7) visual areas involved in disparity processing. We employed an informational connectivity analysis (Coutanche and Thompson-Schill, 2014; Anzellotti and Coutanche, 2018; Koster et al., 2018; Jia et al., 2020) and tested whether these regions shared synchronous discriminability of multivoxel patterns related to disparity processing. Consistent with previous studies (Rockland and Pandya, 1979; Self et al., 2013; Markov et al., 2014), we examined two possible functional connectivity mechanisms (for review: Lawrence et al., 2019b): (1) feedforward processing, as indicated by functional connectivity between superficial and middle layers between earlier and higher visual areas; and (2) feedback processing, as indicated by functional connectivity between deeper layers across areas. We did not test connectivity between superficial layers and deeper layers across areas, as it is known to relate to both feedforward and feedback processing (Fig. 1) (Rockland and Pandya, 1979; Maunsell and van Essen, 1983).

**Figure 6:**
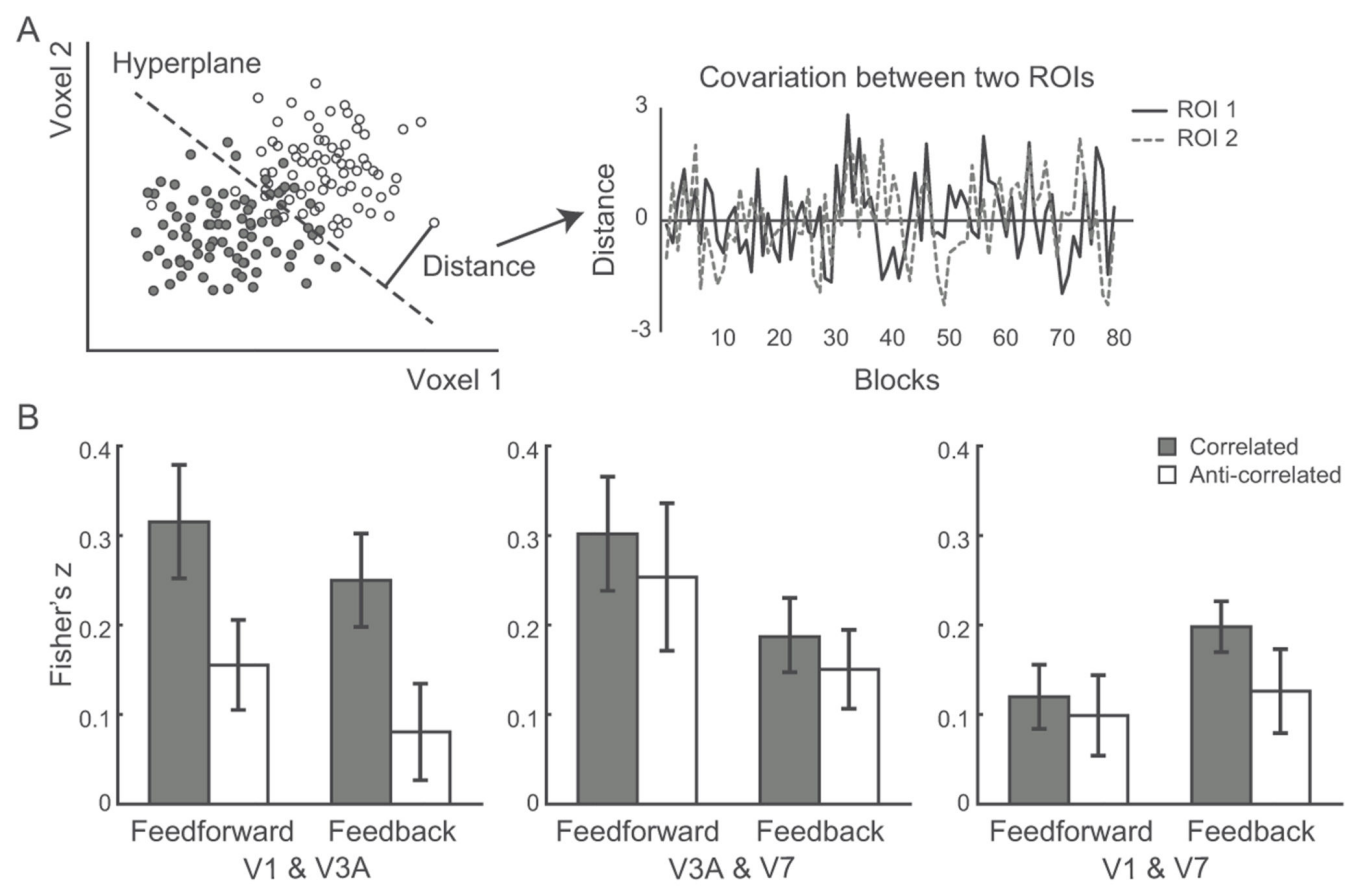
Informational connectivity analysis. (A) Schematic illustration of the procedure followed for the MVPA-based functional connectivity analysis. For each ROI and block, we calculated the distance to the classifier hyperplane (indicated by the dotted line) as index of pattern discriminability (left panel). Filled and open dots indicate test patterns from different classes (i.e., near vs. far disparity). For each ROI, we calculated a time series of distances across blocks during each scanning session. Spearman correlation was used to calculate covariance between time series across ROIs (right panel). (B) Functional connectivity (Fisher’s z) between V1 and V3A; V3A and V7; V1 and V7: feedforward connectivity between superficial layers of earlier visual areas to middle layers of higher areas; feedback connectivity between deeper layers across visual areas for correlated (grey bar) and anti-correlated (white bar) stimuli. Error bars indicate within-subject SEM across participants.

To understand functional connectivity mechanisms that relate to stereoscopic perception, we focused on differences in the information flow for correlated vs. anti-correlated stimuli, rather than overall differences in feedforward vs. feedback processing. We reasoned that stronger connectivity for correlated stimuli supports stereoscopic perception and tested whether this functional connectivity involves feedback vs. feedforward processes. To this end, we tested differences in functional connectivity between correlated vs. anti-correlated stimuli in a) feedforward and b) feedback pathways. Following previous studies employing an informational connectivity analysis (Koster et al., 2018; Jia et al., 2020), we interrogated the MVPA classifiers for each layer and extracted the distance from the hyperplane for the mean pattern signal per block. For each layer per ROI we generated a time course of distance values across blocks and calculated the Spearman correlation between layers across blocks for correlated vs. anti-correlated stimuli (Fig. 6A). Our results showed that functional connectivity for correlated vs. anti-correlated stimuli involves: (1) feedback processing from V3A to V1, and V7 to V3A, while (2) feedforward processing between V3A and V7.

In particular, we showed higher feedforward connectivity between V3A and V7 for correlated than anti-correlated stimuli. A repeated-measures ANOVA (condition × ROI pair) on the correlation coefficients (Fisher’s z) showed a significant main effect of condition (F(1,6) = 9.47, *p* = 0.022, Fig. 6B), main effect of ROI pair (F(2,12) = 8.24, *p* = 0.006), and a significant condition × ROI pair interaction (F(2,12) = 3.92, *p* = 0.049). Post-hoc comparison showed higher connectivity for correlated than anti-correlated stimuli between superficial layers of V3A and middle layers of V7 (t(6) = 6.46, *p* = 0.001), suggesting that signals related to stereoscopic perception are computed in V3A and fed forward to V7. No significant differences were observed in feedforward connectivity between V1 and V3A (t(6) = 1.35, *p* = 0.222) nor between V1 and V7 (t(6) = −0.51, *p* = 0.630).

Further, we showed higher feedback connectivity between V3A and V1, and V7 and V3A for correlated than anti-correlated stimuli. A repeated-measures ANOVA (condition × ROI pair) on the correlation coefficients (Fisher’s z) showed significant main effect of condition (F(1,6) = 8.31, *p* = 0.028, Fig. 6B) and a significant condition × ROI pair interaction (F(2,12) = 6.76, *p* = 0.011), but no significant main effect of ROI pair (F(1.1,6.5) = 0.29, *p* = 0.627). Post-hoc comparison showed: 1) higher connectivity for correlated than anti-correlated stimuli between deeper layers of V1 and V3A (t(6) = 2.76, *p* = 0.032), suggesting that signals related to stereoscopic perception are computed in V3A and fed back to primary visual cortex; and 2) higher connectivity for correlated than anti-correlated stimuli between deeper layers of V3A and V7 (t(6) = 5.03, *p* = 0.003), suggesting that signals related to stereoscopic perception in V7 and fed back to V3A. No significant differences were observed in feedback connectivity between V1 and V7 (t(6) = −0.5, *p* = 0.635).

## Discussion

Here, we capitalize on the sub-millimetre resolution of 7T laminar fMRI to interrogate the circuit processes (feedforward, feedback) that underlie 3D perception. Our results indicate that area V3A is a key nexus for the processing of stereoscopic signals that are propagated to higher dorsal visual areas (V7) and then fed back to primary visual cortex to support 3D perception.

Combining 7T imaging with MVPA, we demonstrate a role of dorsal areas (V3A, V7) in 3D perception, consistent with previous fMRI studies showing a) disparity-evoked fMRI responses in V1, V3A, and V7 (Bridge and Parker, 2007; Preston et al., 2008; Goncalves et al., 2015; Rideaux and Welchman, 2019; Nasr and Tootell, 2020), b) discriminable multivoxel fMRI pattern responses to disparity-defined position (i.e., near vs. far planes) in V3A (Preston et al., 2008; Goncalves et al., 2015; Patten and Welchman, 2015; Li et al., 2017; Liu et al., 2020). Our fMRI results extend beyond these previous studies by demonstrating layer-specific representations related to stereoscopic perception in V3A and V7 (i.e., higher MVPA accuracy for correlated than anti-correlated stimuli in upper than deeper layers). Despite the relatively small sample size (N = 8) in this study, a series of control analyses corroborate our results showing that they are not dependent on methodological choices (e.g., V7 definition, classification methods for decoding, voxel pattern size).

We interpret our results within a framework of feedforward and feedback connectivity across cortical depths (Fig. 1A), as proposed by previous UHF imaging studies (e.g., Kok et al., 2016; Huber et al., 2017; Sharoh et al., 2019; Moerel et al., 2020). In particular, sensory inputs are known to enter the cortex at the level of the middle layer (layer 4) and output information is fed forward through the superficial layers (layer 2/3). In contrast, feedback information is thought to be exchanged between a) deeper layers between visual areas (layer 5/6), b) deeper layers in higher visual areas and superficial layers in lower visual areas (Larkum, 2013; Markov et al., 2014). Previous work has shown that synaptic input to superficial layers may be due to increase in feedback signals carried by neurons that have dendrites projecting to the superficial layers and their cell bodies in deeper layers (Larkum, 2013). Recent UHF brain imaging studies in animals and humans provide converging evidence for this information flow profiles across species (Huber et al., 2021; Jung et al., 2021). Further, neurophysiological studies have shown that this micro-circuit is involved in a range of visual recognition (Self et al., 2013; van Kerkoerle et al., 2014) and attention (Buffalo et al., 2011) tasks. Recent laminar fMRI studies provide evidence for the involvement of this circuit in the context of sensory processing (De Martino et al., 2015; Muckli et al., 2015; Gau et al., 2020; Jia et al., 2020; Zamboni et al., 2020) and visual attention (Fracasso et al., 2016; Scheeringa et al., 2016; Lawrence et al., 2019a). Thus, our results showing layer-specific representations related to stereoscopic perception in upper rather than deeper layers of V3A and V7 suggest that these regions are involved in the processing of input disparity signals and their read out in support of 3D perception.

Next, we interrogated the layer-to-layer functional connectivity between V3A and earlier (i.e., V1) or higher (i.e., V7) visual areas that allows us to test feedforward and feedback processing based on known anatomical connectivity models (Self et al., 2013; Markov et al., 2014; Lawrence et al., 2019a). First, we demonstrate stronger feedforward connectivity for correlated stimuli between superficial layers of V3A and middle layers of V7 and feedback connectivity between deeper layers in these areas. These results suggest that processing of disparity-specific information in V3A involves both feedforward and feedback processes that contribute to 3D perception. These results are consistent with pervious monkey electrophysiology studies showing a functional link between V3A and a homologue region to human V7 (Nakamura et al., 2001) for processing disparity-defined stimuli. Further work, using electrophysiology methods that afford high temporal resolution, is necessary to understand the timings of feedforward and feedback processes and how they contribute to 3D perception.

Second, we demonstrate feedback connectivity specific to stereoscopic perception (i.e., stronger connectivity for correlated than anti-correlated stimuli) between deeper V1 and V3A layers, suggesting top-down influences to disparity processing in primary visual cortex through feedback from higher visual dorsal areas known to be involved in stereo vision and disparity (Goncalves et al., 2015; Nasr and Tootell, 2020). Previous neurophysiology studies have shown attenuated responses in V1 for anti-correlated compared to correlated stimuli (Cumming and Parker, 1997; also in circuit simulation: Samonds et al., 2013). Further, disparity tuning curves of many neurons that respond to anti-correlated stimuli have been shown to be inverted and attenuated compared to the typical responses to correlated stimuli, a possible suppression mechanism in V1 that aids 3D perception (Goncalves and Welchman, 2017). Our functional connectivity results suggest that stronger neural responses for correlated than anti-correlated stimuli in V1 may reflect feedback from higher dorsal visual areas (i.e., V3A) to deeper V1 layers. Previous fMRI studies (Bridge and Parker, 2007; Preston et al., 2008) at standard resolution do not report differences in fMRI responses to correlated vs. anti-correlated stimuli in V1. In contrast, UHF imaging affords the sub-millimetre resolution to capture these differences using MVPA and reveals the fine-scale processes (i.e., feedback) that contribute to 3D perception.

These findings advance our understanding of the mechanisms involved in 3D perception at a finer scale by suggesting a flow of information between primary visual cortex and higher portions of the dorsal visual cortex involved in processing stereoscopic stimuli. While it is clear that anti-correlated stimuli drive responses in primary visual cortex (Cumming and Parker, 1997), responses are attenuated which may reflect the influence of feedback signals from higher visual processing (e.g., V3A). Individual anti-correlated signals per se may be useful in supporting perceptual judgments (Goncalves & Welchman, 2017); however it is clear that a whole display of aRDS does not provide any stable impression of 3D structure, which may drive feedback signals to modulate the activity of disparity-selective neurons in V1 that represent the first stage of binocular processing in the visual system. The use of time-resolved techniques (e.g., MEG, EEG) in addition to laminar specificity may help unpick the functional role of this activity in future work.

It is important to note that, despite the advances afforded by UHF imaging, GE-EPI remains limited by vasculature-related signals contributing to BOLD at the cortical surface, resulting in loss of spatial specificity (Kashyap et al., 2018; Kay et al., 2019). To reduce this superficial bias, we removed voxels with low tSNR (Olman et al., 2007) and high t-statistic for stimulation contrast (Polimeni et al., 2010; Kashyap et al., 2018). Further, we applied a signal unmixing method (Kok et al., 2016; Koster et al., 2018) as a control analysis for draining vein effects from middle to superficial layers. We have previously shown that these corrections return BOLD signal across cortical depths comparable to that recorded using a 3D Gradient And Spin Echo (GRASE) sequence that is known to be sensitive to signals from small vessels and less affected by larger veins, resulting in higher spatial specificity of the measured BOLD signal (e.g., De Martino et al., 2013; Kemper et al., 2015; Zamboni et al., 2020). Finally, we compared BOLD signals across stimulus conditions and cortical depths after z-scoring the signals within each cortical depth to account for possible differences in signal strength across cortical layers (Goense et al., 2012; Havlicek and Uludağ, 2020). Following these corrections, we observed layer-specific differences in MVPA accuracy for correlated compared to anti-correlated stimuli, suggesting that our results are unlikely to be confounded by vasculature-related superficial bias. Our results are consistent with previous laminar imaging studies showing BOLD effects in superficial layers in a range of tasks (De Martino et al., 2015; Muckli et al., 2015; Lawrence et al., 2019a; Gau et al., 2020; Jia et al., 2020) and could not be simply attributed to differences in attention due to task difficulty, as participant engaged in an attentionally demanding task across conditions. Although we used a series of analyses to control for the superficial bias, it could not be completely ruled out. Vascular space occupancy contrast (i.e., VASO) is sensitive to arteriole and post-arterial cerebral blood volume (CBV) changes and shows reduced draining vein contamination compared to BOLD (e.g., Huber et al., 2019; Beckett et al., 2020). The lower functional contrast (SNR) of VASO compared to GE EPI imaging means that longer data acquisition sessions are necessary, that may not always be possible within the timing constraints of human brain imaging studies. However, future work could capitalize on advances in CBV imaging using VASO to enhance the spatial specificity of laminar brain imaging and to control for the superficial bias at the data acquisition stage.

In sum, exploiting UHF fMRI, we provide evidence for the role of V3A in a circuit of feedforward and feedback interactions that support the processing of perceived binocular depth in the visual cortex. Interrogating these interactions at the finer resolution afforded by UHF imaging, we provide the first insights in bridging the gap between animal neurophysiology and human fMRI studies investigating cross-scale circuits: from micro-circuits to global brain networks for 3D perception.

## Significance Statement

Binocular vision plays a significant role in supporting our interactions with the surrounding environment. The fine-scale neural mechanisms that underlie the brain’s skill in extracting 3D structure from binocular signals are poorly understood. Here, we capitalize on recent advances in ultra-high field (UHF) functional imaging to interrogate human brain circuits involved in 3D perception at sub-millimetre resolution. We provide evidence for the role of area V3A as a key nexus for disparity processing that is implicated in feedforward and feedback signals related to the perceptual estimation of 3D structure from binocular signals. These fine-scale measurements help bridge the gap between animal neurophysiology and human fMRI studies investigating cross-scale circuits: from micro-circuits to global brain networks for 3D perception.
